# INMUNOCAT study: The impact of molecular diagnosis on immunotherapy prescription in pollen polysensitized patients from Catalonia

**DOI:** 10.1002/clt2.12246

**Published:** 2023-05-03

**Authors:** Teresa Garriga‐Baraut, M. M. San Miguel Moncín, Mercè Tena, Moisés Labrador‐Horrillo, Comitè d’Al·lèrgia Respiratòria de Catalunya, Spain, O. Asensio, J. Bartra, J. Belmonte, I. Bobolea, C. De Linares, L. Farrarons, S. Miquel, R. Muñoz‐Cano, C. Padró‐Casas, C. Pedemonte, E. Raga, M. Viñas

**Affiliations:** ^1^ Hospital Universitario Vall d’Hebron Barcelona Spain; ^2^ Pius Hospital de Valls Hospital del Vendrell Tarragona Barcelona Spain; ^3^ Thermo Fisher Scientific Barcelona Spain

**Keywords:** asthma, change, immunotherapy, molecular diagnosis, personalized medicine, pollen polysensitized patients, rhinitis, rhinoconjunctivitis

## Abstract

**Background:**

Recognition of specific allergens triggering immune response is key for the appropriate prescription of allergen‐specific immunotherapy (SIT). This study aimed at evaluating the impact of using the commercially available microarray ImmunoCAP^TM^ ISAC 112 (Thermo Fisher Scientific) on the etiological diagnosis and SIT prescription compared to the conventional diagnostic methods in patients with allergic rhinitis/rhinoconjunctivitis and/or asthma.

**Methods:**

300 patients with respiratory allergic disease, sensitized to three or more pollen aeroallergens from different species, as assessed by a skin prick test (SPT) and specific IgE assays (sIgE), were included in this multicentric, prospective observational study. SPT and a blood test were performed to all patients. Total serum IgE and sIgE (ImmunoCAPTM) for allergens found positive in the SPT and sIgE allergen components (ImmunoCAPTM ISAC 112) were measured.

**Results:**

According to SPT results, the most prevalent pollen sensitizers in our population were *Olea europaea* followed by grass, *Platanus acerifolia* and *Parietaria judaica*. The molecular diagnosis (MD) revealed Ole e 1 as the most prevalent pollen sensitizer, followed by Cup a 1, Phl p 1, Cyn d 1, Par j 2, Pla a 1, 2, and 3 and Phl p 5. Immunotherapy prescription changed, due to MD testing, in 51% of the cases, with an increase of prescription of SIT from 39% to 65%.

**Conclusion:**

The identification of the allergen eliciting the respiratory disease is essential for a correct immunotherapy prescription. The advances in allergen characterization using methods, such as the commercial microarray ImmunoCAP^TM^ ISAC 112, can help clinicians to improve SIT prescription.

## 1 INTRODUCTION

Catalonia is a region located in the northeast of the Iberian Peninsula (south of Europe) bathed by the Mediterranean Sea. According to the official pollen information website of the Spanish Society of Allergy and Clinical Immunology (SEAIC) (www.polenes.com), grasses are the most relevant allergy triggering pollens in Spain.^1^ Although sensitization to grass is dominant in the center and north of the peninsula, in the Mediterranean coast, *Parietaria judaica* shifts grasses to the second place, while in the south of Spain, *Olea europaea* is the most common allergy‐triggering pollen, especially in areas with large olive groves. Other important allergenic pollens are weeds, such as *Plantago spp*., *Artemisia vulgaris*, *Salsola Kali* and *Chenopodium album*. At a local level, *Betulaceae* in the north of Spain and *Platanus* and *Cupressus* species in Madrid and Barcelona are also considered responsible for respiratory allergies.^1^

Aeroallergy trends in a certain population reflect exposure to pollens and other aeroallergens, such as dust mites, molds or dander.^2^ It has been widely documented that the prevalence of respiratory allergic diseases, such as asthma and rhinitis/rhinoconjunctivitis (RC), has increased considerably over the recent decades.^3^ This increase has been associated to climate change, which is directly related to greater pollen production and allergenicity.^4^ In fact, both profile complexity of IgE sensitized patients and number of polysensitized patients in our daily medical practice have increased.

Allergen‐specific immunotherapy (SIT) is based on therapeutic vaccination with specific allergens and is the only specific etiology‐based treatment for allergic respiratory diseases (RC, asthma).^5^ The identification of the triggering allergen is essential for a correct allergy diagnosis and SIT prescription. However, commercially available allergen extracts are obtained from natural sources and contain a mix of major and minor allergens as well as non‐allergenic elements. Additionally, there is currently a lack of standardization among the different commercial sources. Therefore, the diagnosis may not be totally accurate, especially in polysensitized individuals who are increasing in numbers.^6^

The Component Resolved Diagnosis (CRD) or molecular diagnosis (MD) has been introduced more than 10 years ago in clinical practice and has changed the paradigm of allergy diagnosis especially in polysensitized patients.^7^, ^8^ Though there is still space for improvement, current MD testing methods allow, in many cases, clinicians to determine the exact molecule to which the patient is genuinely sensitized. This has raised some fundamental questions regarding a proper allergy diagnosis and an accurate SIT prescription, as follows: Is it worth to use vaccine extracts that do not contain significant amounts of the molecular component driving patient sensitization? Is it effective to use vaccines against allergens that are not genuine sensitizers? Should MD be always used in patients who are candidates for vaccines? In order to address these questions, it is important to analyze the impact of MD on SIT prescription and in case considerable changes in the prescription patterns are revealed, evaluate if such changes are associated with better patient outcomes. While some research groups have shown that MD significantly modifies SIT prescription,^9^, ^10^, ^11^, ^12^ more studies assessing its impact on vaccination outcomes are warranted.

This paper aims to evaluate the epidemiology of allergy sensitization in Catalonia and to assess/confirm whether MD can modify immunotherapy prescription.

## 2 MATERIALS AND METHODS

### 2.1 Study design and patient selection

This is a multicentric, prospective observational study that recruited a total of 300 consecutive patients from 12 different hospitals across Catalonia, who were previously diagnosed with asthma and rhinitis, according to Spanish guidelines (supplementary Table S1). This study included patients from 3 to 82 years, attending a participating outpatient clinic between 2018 and 2019.

Ethical approval was granted by the Clinical Research Ethics Committee (CEIC) from Vall D’Hebron Institut de Recerca (VHIR) (PR(AMI)244/2013). The study was also approved by the Clinical Research Ethics Committees corresponding to each participant Hospital. All patients (or their parents/guardians in case of children) gave their written informed consent to participate. The experiments were carried out in accordance with the principles of the Declaration of Helsinki.

Both the collection and processing of personal data were performed in accordance with the Spanish Organic Law 3/2018 on the protection of personal data. All personal data and sample references were anonymized using independent codes maintained into a database under secure conditions.

### 2.2 Participants

Inclusion criteria were as follows:

‐ Patients diagnosed with respiratory allergic disease within the previous 2 years: intermittent or persistent, moderate to severe, rhinitis/rhinoconjunctivitis and/or mild or moderate persistent asthma, according to ARIA^13^ and GEMA 4.2^14^ guidelines attending for the first time or for follow‐up visits an outpatient allergology clinic. Subjects were classified as allergic if any of the following applied: (i) a solid history of allergy and a positive sensitization test (SPT) or (ii) a compatible clinical history and positive tests (SPT and/or specific IgE (sIgE) to a whole extract or specific molecules);
‐ Patients sensitized to three or more pollen aeroallergens from different species as confirmed by SPT. Sensitization to other aeroallergens or food allergens was not considered an exclusion criterion.
‐ Subjects living in the same geographical region for at least 2 years;
‐ Patients who agreed to sign an informed consent form. For patients under 18 years of age parents or guardians had to sign the informed consent.

Patients were excluded in case of uncontrolled severe asthma, severe atopic dermatitis, dermographism, or any other baseline disease for which diagnostic testing (SPT) is controversial. Patients who had received previous allergen immunotherapy, those for whom immunotherapy prescription was not indicated and those with neoplastic and/or autoimmunologic diseases were also excluded.

Three patients aged 3, 4 and 82 years were included in the study although their age was outside the classical immunotherapy age range. They were not excluded from the analysis since for the purposes of this study the recommendation for SIT was purely hypothetical and proposed on the basis of detected sensitizations.

Sample size was inferred based on the estimation that 80% of the pediatric, adolescent, and young adult population attending the allergology outpatient clinic has some type of respiratory pathology (rhinitis, RC and/or asthma). Of these, between 30% and 65% are expected to have a positive SPT to three or more allergens.^15^ Based on these data, it was calculated that a minimum of 70 patients would be required to achieve sufficient statistical power.

### 2.3 Variables

Clinical, demographic, and anthropometric variables for each patient participating in the study were collected by each researcher.

SPTs were performed with standardized inhalant allergen extracts used in routine testing in every hospital, including the following pollen aeroallergens^15^, ^16^: *Parietaria judaica*, *Artemisia vulgaris*, *Salsola kali*, *Chenopodium album*, *Plantago lanceolata*, *Betula verrucosa*, *Corylus avellana*, *Platanus acerifolia*, *Cupressus arizonica*, *Olea europaea*, *Phleum pratense*, *Cynodon dactylon*, and *Phragmites australis*. Peach lipid transfer protein (LTP) and palm tree profilin were also included in the SPT. All patients had been instructed not to take medications during the 7 days before the test.

Histamine hydrogen chloride 10 mg/mL was used as positive control and physiologic saline as negative control. All SPTs were conducted using injections in the volar surface of each forearm. SPTs were performed following EAACI recommendations. Papules were measured at 15 min. Papules were considered positive if they were greater than 7.1 mm^2^, which would equal a papule of 3 mm in diameter.

Blood samples were taken from all patients included in the study. The following parameters were measured: total serum IgE; specific IgE to whole allergens detected by SPT (Phadia 250 Laboratory System; Thermo Fisher Scientific); IgEs to specific molecular components (microarray ImmunoCAP^TM^ ISAC 112; Thermo Fisher Scientific, see supplementary Table S2 for complete list of components) to have a wide picture on patient sensitization profiles. A semi quantitative measurement of molecular components was performed using a Luxcan scanner according to the manufacturer's specifications. The results were determined using ISAC standardized units and considered positive if ≥ 0.3 ISU were reported.

### 2.4 Recommended prescription of allergen immunotherapy

All the researchers filled out a questionnaire for each patient including the hypothetical indication and the allergen composition of the SIT that they would (or would not) recommend based on data obtained from clinical history, SPT and specific IgE results before obtaining the results of MD. After obtaining the result of ImmunoCAP^TM^ ISAC 112, the same researcher had to fill out a second copy of the same questionnaire. Determinations were considered in agreement if indication of SIT and selected allergens were the same in the two copies of the questionnaire (i.e., before and after MD results were obtained). In both cases, ITA was recommended according to the main guidelines that were prevailing during the study period.^17^, ^18^, ^19^

### 2.5 Statistical analysis

A descriptive analysis of the study data has been carried out through the elaboration of frequency tables for the nominal type variables and measures of central tendency and dispersion for the continuous variables. The comparison between groups was carried out using Fisher's exact test for qualitative variables. In the case of quantitative variables, the Student's *t* test was performed for the comparison between 2 groups and the ANOVA test for the comparison of 3 or more groups. The ISAC result was presented by subject using a heatmap (based on ISAC classes) and using boxplots by component and by age groups. SIT use before and after MD was presented using a heatmap (use vs. no use). Detailed SIT use of the specific target allergens was presented showing the percent of subjects whose SIT contained each specific allergen both before and after MD, also segmented by age. All calculations were performed using the SAS 9.4 statistical package with a significance limit of 0.05.

## 3 RESULTS

### 3.1 Demographics

A total of 300 patients were included in this study. Table 1 shows the demographics and most relevant clinical data. The average age of the pollen polysensitized population at the time of inclusion was 32.1 years (ranging from 3 to 82 years), 11.7% of whom were children (<11 years); 24.7% were adolescents and young adults (11–25 years) and 63.7% were adults >25 years of age. The most common allergic disease was rhinoconjunctivitis (97.7%), followed by food allergy (49%) and asthma (43%) according to the allergy specialist's final diagnosis. Further details on the food allergy and asthma sensitization profiles of the cohort are shown in supplementary Figures S1 and S2.

**TABLE 1:** Study cohort demographics.

	*n* (%)
Age groups (years old)	
Age <11	35 (11.7)
Age [11; 25]	191 (63.7)
Age >25	74 (24.7)
Gender	
Female	158 (52.7)
Male	142 (47.3)
City of origin	
Data missing	1 (0.3)
Rural	11 (3.7)
Suburban	29 (9.7)
Urban	259 (86.3)
Prevalence of allergic disease	
Anaphylaxis	26 (8.7)
Asthma	129 (43)
Intermittent	75 (58.1)
Persistent	52 (40.31)
Mild	99 (76.74)
Moderate	26 (20.15)
Rhinoconjunctivitis	293 (97.7)
Intermittent	112 (38.2)
Persistent	181 (61.7)
Mild	128 (43.6)
Moderate	148 (50.51)
Severe	14 (4.8)
Atopic dermatitis	63 (21)
Food allergy	147 (49)
Furry animals	91 (30.3)
Urticaria/angioedema	62 (20.7)
Risk factors	
Family history of atopy	154 (51.3)
Toxic habits	34 (11.3)
Passive toxic habits	37 (12.3)

*Note*: Key: *n* = number of patients.

### 3.2 Pollen sensitization profile according to SPT

**FIGURE 1:**
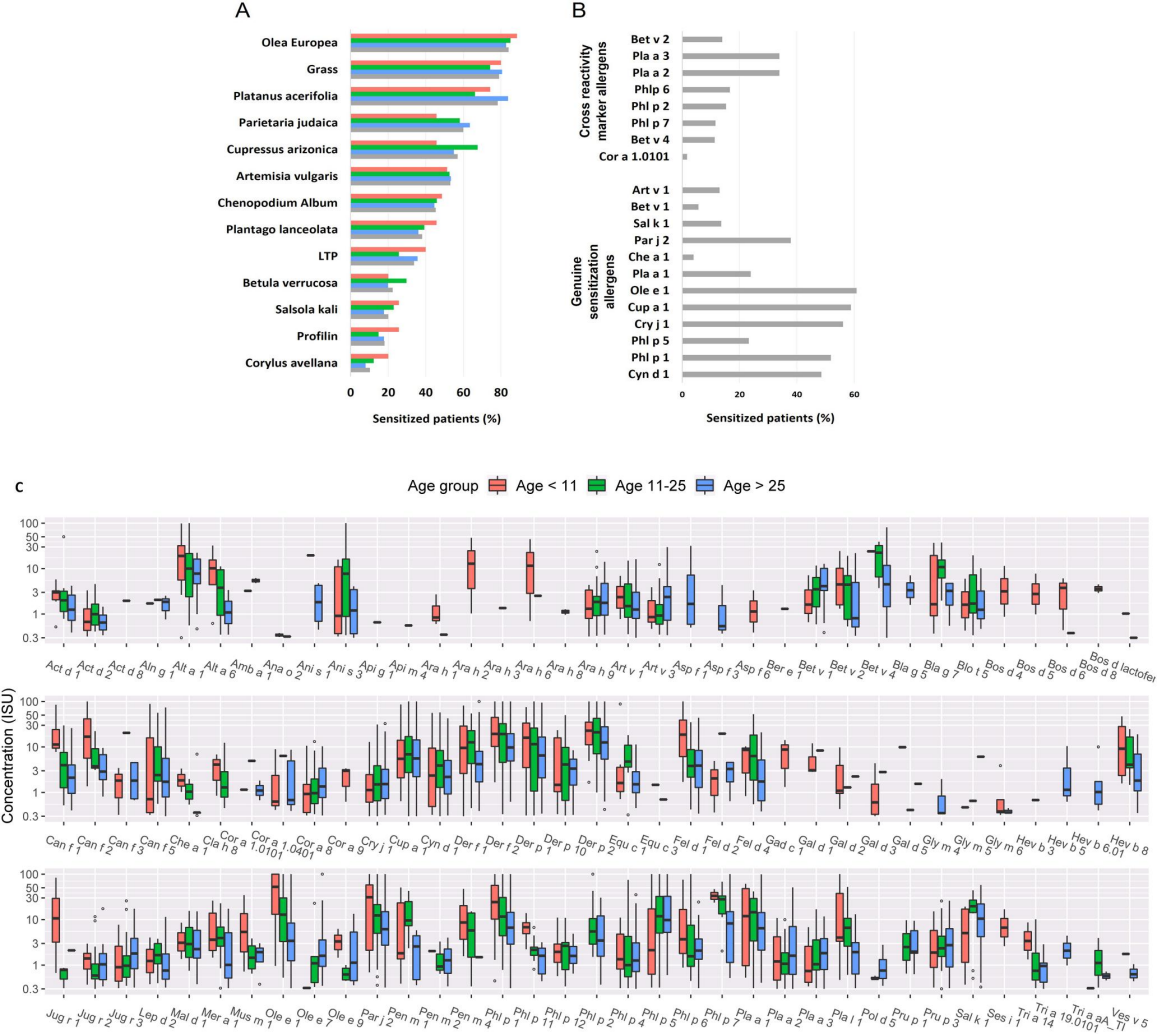
Pollen sensitization profile of the cohort. (A), Frequency of positive reactions to most common allergens across age groups, according to skin prick tests. (B), Frequency of positive reactions to the most common molecular allergens according to ImmunoCAP^®^ ISAC; allergens were classified into: specific allergens (leading to genuine sensitization) and cross‐reactive allergens. (C), Complete sensitization profile according to ImmunoCAP^®^ ISAC across age groups. Pink: <11 years; green: 11–25 years; blue: >25 years; grey: all subjects.

According to SPT results, the most prevalent sensitizers were *Olea europaea* followed by grass (*Cynodon dactylon + Phleum pratense* + *Phragmites australis*) and *Platanus acerifolia* (Figure 1A).

### 3.3 Pollen sensitization profile according to ImmunoCAP^TM^ ISAC 112

ImmunoCAP^TM^ ISAC 112 results showed a similar sensitization pattern to SPT (Figure 1B,C). Ole e 1 was the most prevalent individual pollen sensitizer, followed by Cup a 1, Cry j 1, Phl p 1, Cyn d 1, Par j 2, Pla a 1, 2, and 3 Phl p 5. Interestingly, Che a 1, Bet v 1, 2 and Cor a 1.01 showed modest ISAC^TM^ 112 results in agreement with the SPT results.

### 3.4 Comparison between ImmunoCAP sIgE and SPT results: Qualitative and quantitative analyses

#### 3.4.1 Qualitative analysis

**FIGURE 2:**
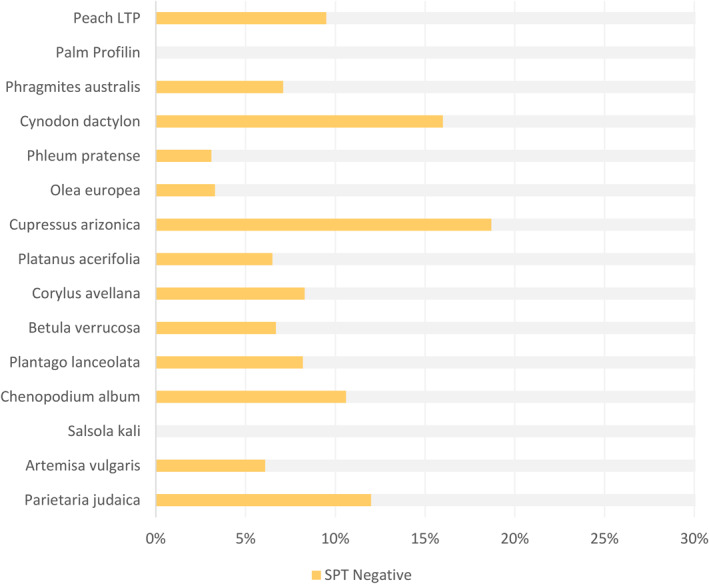
Comparison between ImmunoCAP^®^ sIgE and SPT results for the most common pollen allergens. Graph highlights the frequency of negative SPT in positive ImmunoCAP^®^ sIgE patients. Peach LTP = lipid transfer protein.

An overall average of 7.7% of the patients had negative SPT results while testing positive for sIgE, considering 0.35 kU_A_/l as the cut‐off value for the latter. The highest discrepancies were found for *Cupressus arizonica* (18.7%), *Cynodon dactylon* (16.0%), *Parietaria judaica* (12%), and *Chenopodium album* (10.6%). No discrepancies were found for *Salsola kali* and profilins (Figure 2).

#### 3.4.2 Quantitative analysis

Patients with a negative SPT sometimes showed positive sIgE levels. Quantitatively, we distinguished into four groups: a) patients with negative results to SPT and sIgE, b) patients with negative SPT and sIgE values between 0.1 and 0.35 kU_A_/l (equivocal range), c) patients with negative SPT and sIgE values between 0.35 and 1 kU_A_/l, and d) patients with negative SPT and sIgE values above 1 kU_A_/l. In the first group (negative to both tests), *Salsola kali* and profilins were predominant. In the second group (0.1–0.35 kU_A_/l), *Chenopodioum album*, *Plantago lanceolata*, *Betula verrucosa*, *Corylus avellana*, *Phleum pretense*, *Phragmites australis*, and LTP were mostly found. In the third group *Artemisia vulgaris*, *Platanus acerifolia*, *Cupressus arizonica*, and *Olea europaea* were the predominant pollens. Finally, in the fourth group, *Cynodon dactylon* and *Parietaria Judaica* showed mean values above 1 kU_A_/l.

### 3.5 Immunotherapy prescription

**FIGURE 3:**
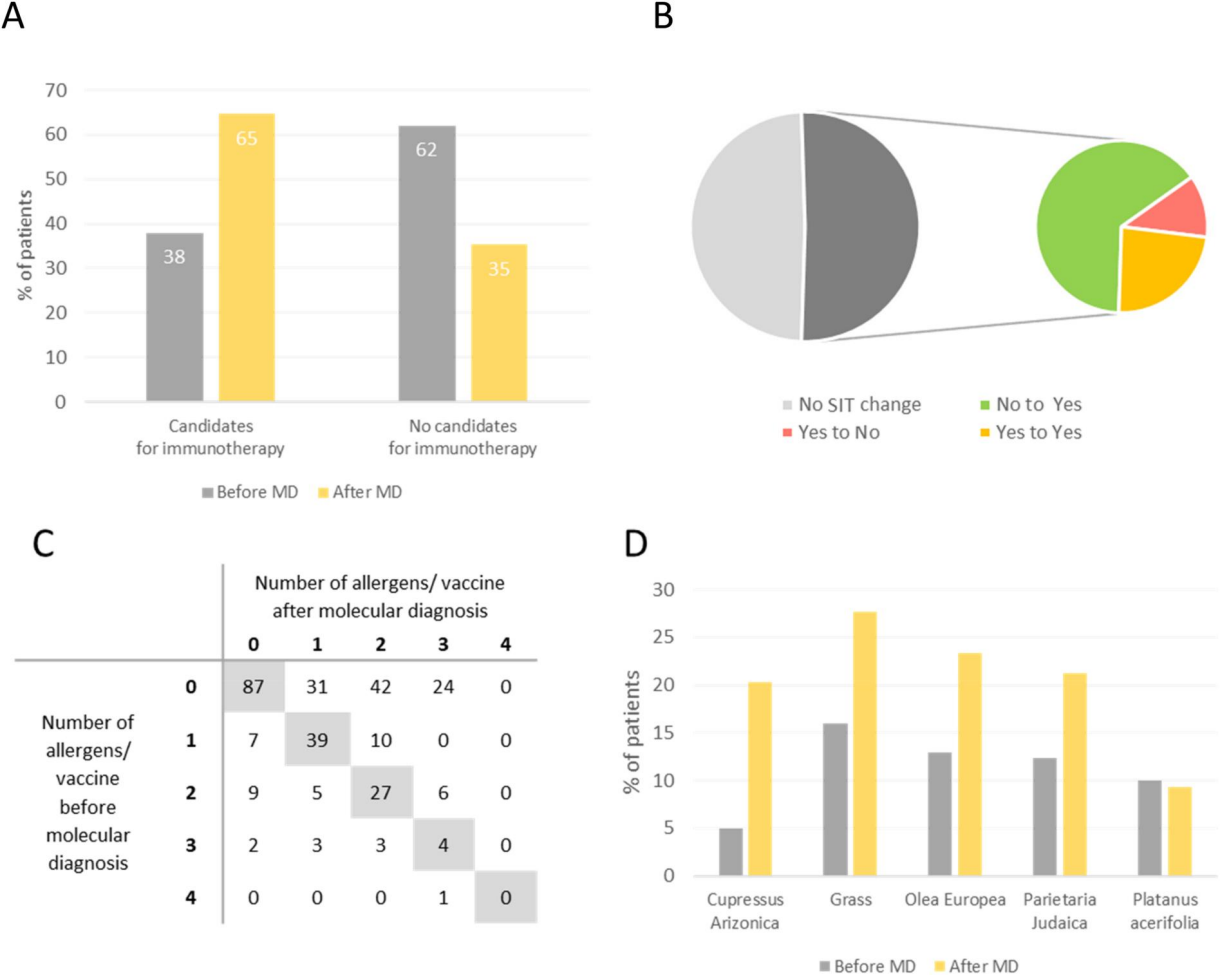
Changes in the immunotherapy (SIT) prescriptions following molecular diagnosis. (A), Percentage of patients who were prescribed SIT (candidates for immunotherapy) versus percentage of patients that were not prescribed SIT (non‐candidates for immunotherapy) before and after molecular diagnosis. (B), Changes in the SIT prescriptions following molecular diagnosis: patients who became eligible for SIT (“No to Yes”: 33%), patients who were no longer considered candidates for SIT (“Yes to “No”: 6%), or patients who were still eligible for SIT, but had their SIT prescription adjusted. (C and D), Adjustments in the SIT prescription in terms of the allergens (C) composition and (D) number.

Immunotherapy prescription changed due to MD testing in 51% of the cases (Figure 3B). The number of patients potentially benefiting from SIT increased from 39% to 65% while the number of patients who were initially not considered as candidates for vaccination decreased (Figure 3A, supplementary Figure S3). More specifically, 32% of the SIT prescriptions changed from no to yes, whereas 6% of the prescriptions switched from yes to no (Figure 3B). Moreover, in occasions where allergy vaccination would have been recommended both prior and after MD, the composition of the vaccine changed in 13% of the cases (supplementary Figure 4). Another important element to consider is that MD also implied a change in the number of whole extracts included in the vaccination plan. While in 52.3% of the prescriptions, the number of selected allergens remained the same, in 37.7% of the cases, the number of allergens included in the composition of the vaccine increased. Only in 10% of the prescriptions, the number of allergens to be included in the vaccine decreased after MD (Figure 3C and supplementary Figure S6).

### 3.6 Immunotherapy prescription by age

The change in SIT prescription followed the same trend across all age groups with half of the prescriptions changing in all age brackets and a clear dominance of a no to yes change [29% (<), 24% (11–25 years) and 36% (>25 years)]. Therefore, a clear increase in SIT prescription was observed after MD (Figure 2 and supplementary Figure S3). The increase was more significant at ages >25 years with a 31.6% increase and a 16.2% increase for patients between 11 and 25 years and a 20% increase for children under 11 years of age. As observed in the general cohort, the rate of the yes to no change was inferior to the rate of no to yes in all age ranges (Figure 3C supplementary Figures S5 and S6). Regarding changes in vaccine composition, the rate was higher for younger patients [14% (<11 years), 19% (11–25 years) and 9% (>25 years)].

The most prevalent pollens were those corresponding to *Cupressus arizonica*, grass species (*Phleum pratense* + *Cynodon dactylon + Phragmites australis)*, *Olea europaea*, *Parietaria judaica*, and *Platanus acerifolia*. With the exception of *Platanus acerifolia*, which had the same prescription levels both before and after MD, all other pollens showed a clear increase in prescription after MD. *Cupressus arizonica* displayed the greatest amount of change (15.3% increase), followed by grass (*Phleum pratense* + *Cynodon dactylon + Phragmites australis)* (11.7%), *Olea europaea* (10.3%), and *Parietaria judaica* (26.2%). This trend was observed in all age ranges (Figure 3D and supplementary Table S3). The specific immunotherapy allergen composition prescribed before and after molecular diagnosis is detailed in supplementary Table S5.

### 3.7 Evaluation of the correlation between rhinoconjunctivitis/asthma severity and the sensitization intensity to specific pollen components

The correlation between ImmunoCAP^TM^ ISAC 112 signal intensity and the severity of the asthma/rhinoconjunctivitis was evaluated considering patients with mild and moderate disease due to the low number of patients suffering from a severe pathology (not enough to reach statistically significant results). A positive correlation was considered for p values less than 0.05. A statistically significant correlation was only found for Sal k 1 (*p* = 0.032) in patients suffering from rhinoconjunctivitis. Regarding asthma, no correlation was found in any of the pollen components analyzed in this study (supplementary Tables S3 and S4 and table supplementary Figure S2).

## 4 DISCUSSION

The main objective of this study was to evaluate the impact of MD on immunotherapy prescription.

To that end, we evaluated the patients' sensitization profiles using three different methods: SPT, ImmunoCAP^TM^ sIgE, and ImmunoCAP^TM^ ISAC 112. According to all methods, *Olea europaea* was the main sensitizing pollen, although, interestingly, *Olea europaea* is not extensively grown in Catalonia. In general terms, the 3 tests agreed in terms of the sensitization prevalence for each pollen although some discrepancies were found (e.g., *Chenopodium album* and Che e 1 and *Artemisia vulgaris*, Art v 1 and Art v 3). Such discrepancies can be explained by the fact that whole extract sensitization values cannot be directly compared to values of sensitization to specific molecular components as other constituents of the whole extract can contribute to sensitization.^8^

To determine the usefulness of SPT as a first‐line screening tool for polysensitized patients, we qualitatively and quantitatively compared the test results with the corresponding serum‐specific IgE to whole allergens (ImmunoCAP^TM^) as it is considered the reference method. Discrepancies between tests have already been described.^20^ About 18.7% of patients with a negative SPT test for *Cupressus arizonica* were found positive in the ImmunoCAP^TM^ test, highlighting an important degree of underdiagnosis for the fourth most important sensitizer in our population. A similar situation was found for *Cynodon dactylon* (16.0%), *Parietaria judaica* (12.0%), and *Chenopodium album* (10.6%). At a quantitative level, *Artemisia vulgaris*, *Platanus acerifolia*, *Cupressus arizonica*, and *Olea europaea* were positive in patients with negative SPTs. Moreover, *Cynodon dactylon* and *Parietaria judaica* showed a discrepancy between SPT and whole IgE testing in more than 10% of the patients with an average value above 1 kU_A_/l, indicating an important degree of misdiagnosis from SPT for these particular allergens.

Despite the general agreement among testing methods with respect to the main allergenic pollens, many discrepancies have been found between SPT and MD methods (see above), which are in agreement with other publications.^21^, ^22^ Therefore, MD has a significant impact on SIT prescription in our region at three different levels: changing the SIT composition, helping identify new candidates for SIT, and revealing that some patients who would have been recommended SIT based on SPT results were not good candidates after all. In fact, SIT prescription changed due to MD for 51% of the participating patients, matching previous reported data.^10^ 32% of the patients who were originally excluded from SIT were considered good candidates based on the MD results, giving these patients an option to improve their quality of life. On the other hand, 6% of patients initially prescribed SIT were not considered adequate candidates, allowing savings for the healthcare system without detriment to patients' health. It is remarkable that, in 12% of patients who were prescribed SIT, the composition changed based on MD. This was mainly translated to an increase in the number of components, probably due to the identification of more sensitization profiles corresponding to a mix of true sensitizations instead of a single genuine sensitization profile with a high degree of cross‐reactivity. Vaccines including 1 or 2 components have been described to be beneficial.^23^ However, in our population, 34 patients (11.3%) had a final prescription of a 3‐component vaccine, while no patient received a prescription for a 4‐component vaccine, as it is not recommended. For vaccines including a mix of three or more non‐related pollen allergens, there is still an unmet need for efficacy evaluation, which explains their low prescription rate.^24^ The impact of MD on SIT followed the same trend when results were analyzed by age ranges (pediatric, adolescent and young adult, and adult patients) with prescription changes in half of the patients in each age range.

These results disagree with data published by Saltabayeva et al. who studied 95 patients with allergic rhinitis and reported a decrease in the number of SIT prescription after MD (119 SIT treatments) as compared to SPT (275 treatments). However, an important limitation of this study was that only 6 molecules were analyzed: nArt v 1, rArt v 3, rAmb a 1, rPhl p 1, rPhl p 5, and rBet v 1.^22^ However, what we observed was that if only SPT results are considered in order to decide which molecules should be further studied as potential allergens, a bias in the diagnosis could be easily produced due to the diagnostic limitations of the SPT.

An earlier work published by Sastre et al.^25^ is the only publication we have identified regarding the evaluation of SIT prescription using MD to complement SPT. This study performed in Madrid (Spain) included 141 patients (average age 31 ± 13 years) with allergic RC and/or asthma, sensitized to pollen. Sastre et al. used ISAC^TM^ (focusing on 96 allergens) and reported that MD led to prescription changes in 54% of the studied population. They found low agreement comparing SPT and ISAC^TM^, especially regarding *Platanus acerifolia*, Pla a 1 and Pla a 2. These data highlight the usefulness of using multiparametric tests to overcome diagnostic limitations of the SPT.

There are two main limitations of our study. On one hand, the lack of evaluation of the clinical outcomes of the new composition of SIT prescription following MD. Nevertheless, it has to be taken into account that in this study, we set out to evaluate the hypothetical prescription of SIT. The evaluation of the correlation with clinical outcomes of the SIT is the next step and a key point for the appropriate management of pollen polysensitized patients. On the other hand, we are evaluating a specific cohort that does not represent the entire allergic population in our area of study, as only patients sensitized to 3 or more pollens were considered and more than half of the included patients live in Barcelona and its metropolitan area.

## 5 CONCLUSIONS

The use of MD significantly impacts, probably by increasing accuracy, SIT prescription patterns by allergologists, not only by increasing the number of patients considered good candidates for vaccination but also by changing SIT composition in patients prescribed SIT prior to molecular diagnosis. Our study reveals that in our cohort, sensitization to some pollens is underdiagnosed by SPT, so although SPT is a good first‐line screening approach, if there are compatible clinical symptoms, the results should be complemented with the determination of sIgE to whole allergens. Also, for the same pollen, SIT is underprescribed if MD is not used for diagnosis. The clearest example in our cohort is allergy to *Cupressus arizonica*, a really important pollen in the Mediterranean region, which is clearly underdiagnosed and thus undervaccinated. This study highlights the importance of sIgE at all levels (total IgE, whole allergens, and molecular components) for appropriate allergy diagnosis and treatment.

## AUTHOR CONTRIBUTIONS

**Teresa Garriga‐Baraut**: conceptualization (equal); data curation (equal); formal analysis (equal); funding acquisition (equal); investigation (equal); methodology (equal); supervision (equal); validation (equal); writing – original draft (equal); writing – review and editing (equal). **M. M. San Miguel Moncín**: conceptualization (equal); data curation (equal); formal analysis (equal); funding acquisition (equal); investigation (equal); methodology (equal); project administration (equal); supervision (equal); validation (equal); writing – original draft (equal); writing – review and editing (equal). **Mercè Tena**: formal analysis (equal); writing – original draft (equal); writing – review and editing (equal). **Moisés Labrador‐Horrillo**: conceptualization (equal); data curation (equal); formal analysis (equal); funding acquisition (equal); investigation (equal); methodology (equal); supervision (equal); validation (equal); writing – original draft (equal); writing – review and editing (equal).

## CONFLICT OF INTEREST STATEMENT

Mercè Tena works as Scientific Liaison at Thermo Fisher Scientific. The remaining authors have no conflicts of interest to declare.

## Supporting information

Supporting Information S1
