# Supplementary material for: INMUNOCAT study: The impact of molecular diagnosis on immunotherapy prescription in pollen polysensitized patients from Catalonia
Source: Clin Transl Allergy. 2023 May 3;13(5):e12246. doi: 10.1002/clt2.12246 (PMC10154984; doi:10.1002/clt2.12246)
Supplement: Supplementary file 1 — Supporting Information S1 [file CLT2-13-e12246-s001.docx]

SUPPLEMENTARY MATERIAL

Supplementary table 1. Hospitals that participated in the study

| **Hospital** | **Region** |
| --- | --- |
| Hospital General de Catalunya | Sant Cugat |
| Hospital de Nens de Barcelona | Barcelona |
| Hospital Germans Trias i Pujol | Badalona |
| Hospital Universitari Parc Taulí | Sabadell |
| Hospital de Sant Joan de Déu | Manresa |
| Hospital Joan XXIII | Tarragona |
| Hospital Clínic de Barcelona | Barcelona |
| Hospital de Terrassa | Terrassa |
| Hospital de Tortosa Verge de la Cinta | Tortosa |
| Hospital Mútua de Terrassa | Terrassa |
| Centro Médico Téknon | Barcelona |
| Pius Hospital de Valls | Valls |
| Hospital Universitario Vall d'Hebron | Barcelona |

Supplementary table 2. Allergen components included in ImmunoCAP^TM^ ISAC 112

| Act d 1 | Ara h 3 | Bla g 7 | Cor a 9 | Gal d 1 | Mal d 1 | Phl p 5 |
| --- | --- | --- | --- | --- | --- | --- |
| Act d 2 | Ara h 6 | Blo t 5 | Cry j 1 | Gal d 2 | Mer a 1 | Phl p 6 |
| Act d 5 | Ara h 8 | Bos d 4 | Cup a 1 | Gal d 3 | Mus m 1 | Phl p 7 |
| Act d 8 | Ara h 9 | Bos d 5 | Cyn d 1 | Gal d 5 | MUXF3 | Pla a 1 |
| Aln g 1 | Art v 1 | Bos d 6 | Der f 1 | Gly m 4 | Ole e 1 | Pla a 2 |
| Alt a 1 | Art v 3 | Bos d 8 | Der f 2 | Gly m 5 | Ole e 7 | Pla a 3 |
| Alt a 6 | Asp f 1 | Bos d lactoferrin | Der p 1 | Gly m 6 | Ole e 9 | Pla l 1 |
| Amb a 1 | Asp f 3 | Can f 1 | Der p 10 | Hev b 1 | Par j 2 | Pol d 5 |
| Ana o 2 | Asp f 6 | Can f 2 | Der p 2 | Hev b 3 | Pen m 1 | Pru p 1 |
| Ani s 1 | Ber e 1 | Can f 3 | Equ c 1 | Hev b 5 | Pen m 2 | Pru p 3 |
| Ani s 3 | Bet v 1 | Can f 5 | Equ c 3 | Hev b 6.01 | Pen m 4 | Sal k 1 |
| Api g 1 | Bet v 2 | Che a 1 | Fag e 2 | Hev b 8 | Phl p 1 | Ses i 1 |
| Api m 1 | Bet v 4 | Cla h 8 | Fel d 1 | Jug r 1 | Phl p 11 | Tri a 14 |
| Api m 4 | Bla g 1 | Cor a 1.0101 | Fel d 2 | Jug r 2 | Phl p 12 | Tri a 19.0101 |
| Ara h 1 | Bla g 2 | Cor a 1.0401 | Fel d 4 | Jug r 3 | Phl p 2 | Tri a aA_TI |
| Ara h 2 | Bla g 5 | Cor a 8 | Gad c 1 | Lep d 2 | Phl p 4 | Ves v 5 |

Supplementary table 3. Sensitization frequencies corresponding to the included patients before and after MD to the most relevant pollens. Results are stratified by age.

|  |  | *Cupressus arizonica* | *Grass* | *Olea europea* | *Parietaria judaica* | *Platanus acerifolia* |
| --- | --- | --- | --- | --- | --- | --- |
| General | Before MD (%) | 5 | 16 | 13 | 12.3 | 10 |
|  | After MD (%) | 20.3 | 27.7 | 23.3 | 21.3 | 9.3 |
| < 11 | Before MD (%) | 2.9 | 25.7 | 17.1 | 5.7 | 8.6 |
|  | After MD (%) | 11.4 | 31.4 | 37.1 | 5.7 | 8.6 |
| 11 to 25 | Before MD (%) | 2.7 | 16.2 | 13.5 | 13.5 | 10.8 |
|  | After MD (%) | 16.2 | 17.6 | 16.2 | 16.2 | 6.8 |
| > 25 | Before MD (%) | 6.3 | 14.1 | 12 | 13.1 | 9.9 |
|  | After MD (%) | 23.6 | 30.9 | 23.6 | 26.2 | 10.5 |

Supplementary figure 1: Food sensitization profile of pollen polysensitized patients in Catalonia. Frequency of positive reactions to most common allergens across age groups according to skin prick testing.

Supplementary figure 2: Asthma severity association to aeroallergen sensitization intensity according to ImmunoCAP ISAC results.

Supplementary figure 3. Overview of ITA change due to DM.


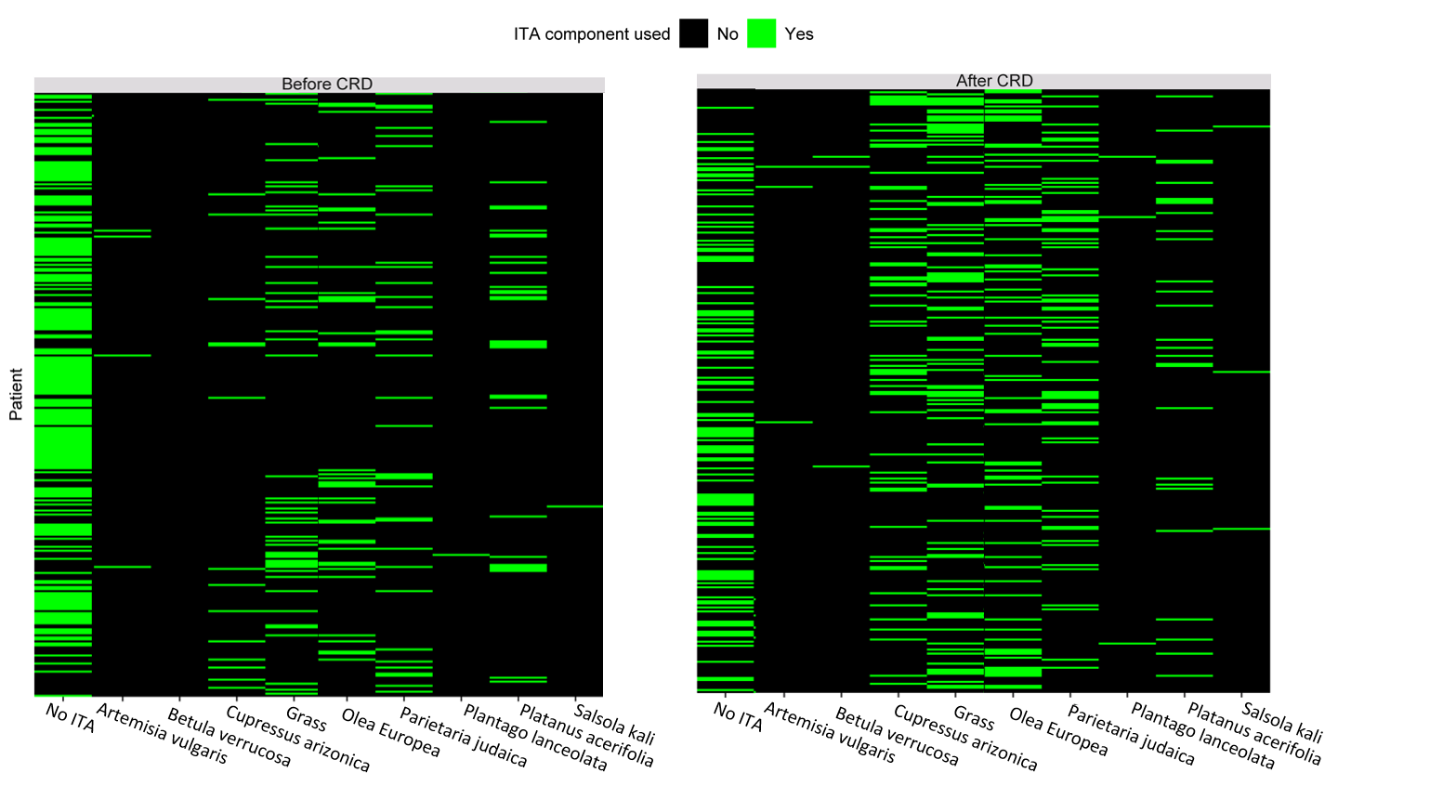


Supplementary figure 4. Overview of ITA change according to the typo of change (Yes:Yes, No:Yes, Yes:No, No:No)


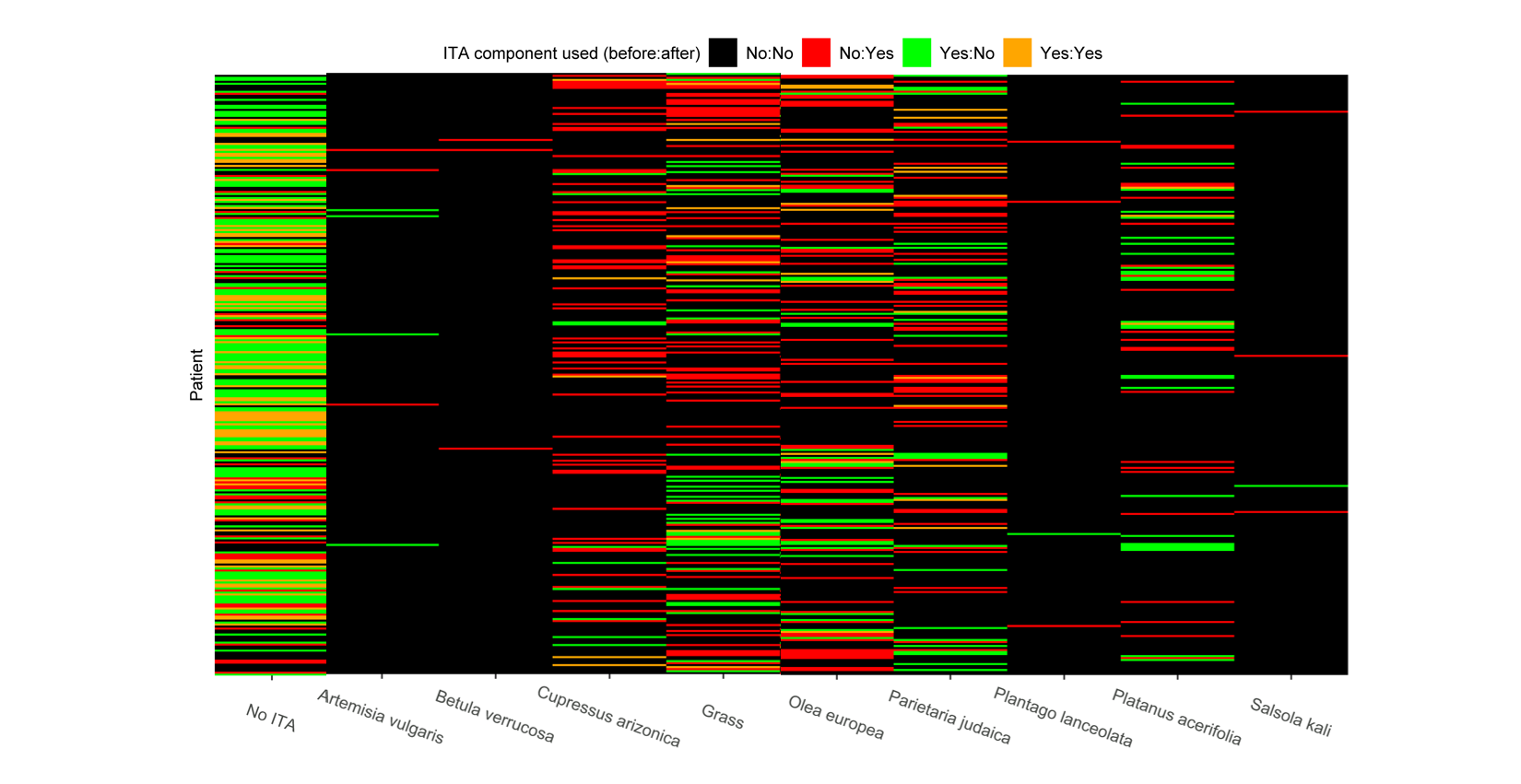


Supplementary figure 5. ImmunoCAP ISAC heatmap per age range.
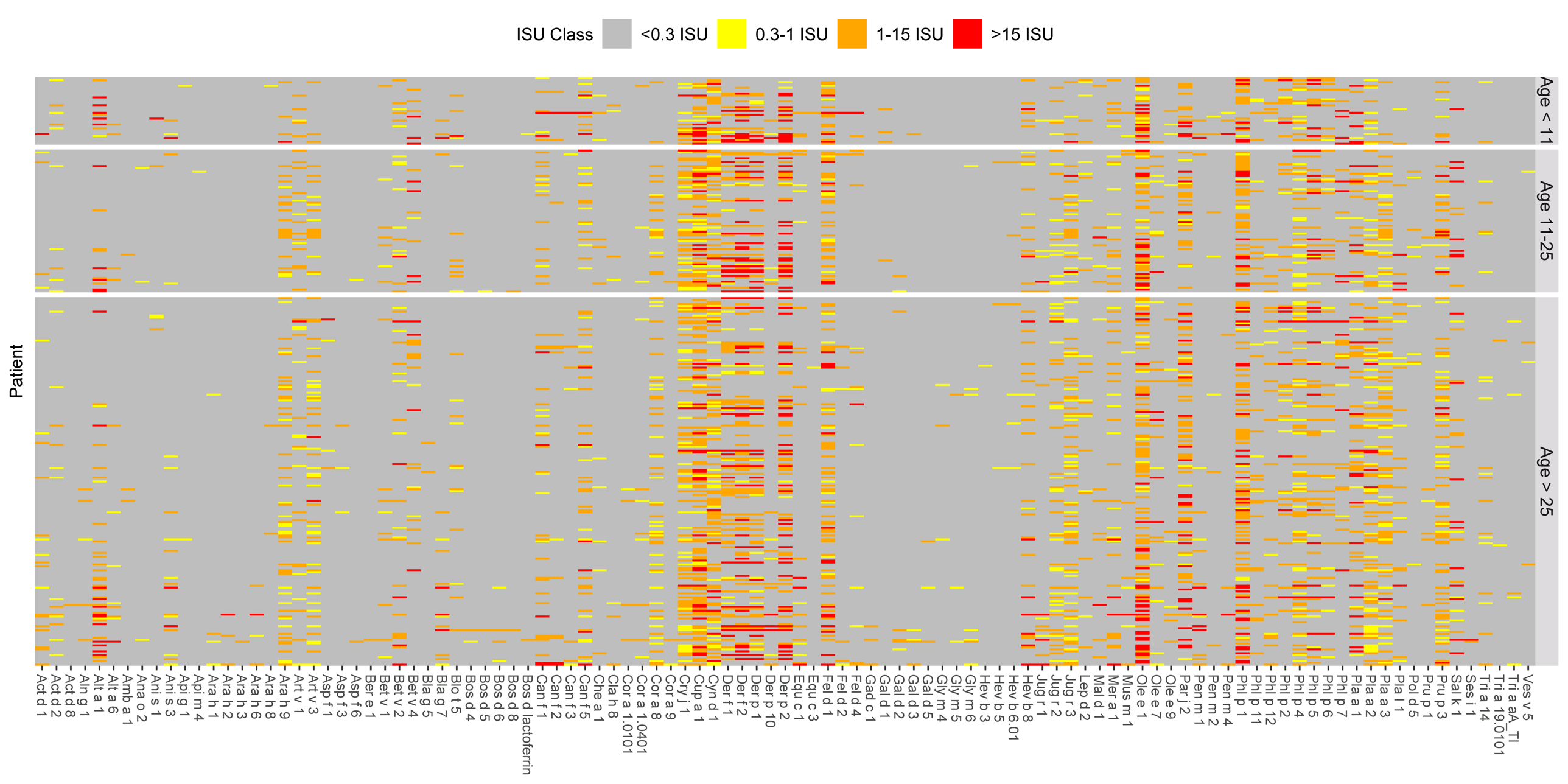


Supplementary figure 6. Changes in SIT prescription following molecular diagnosis by age range. (A) SIT prescription change before and after MD split by age range. (B) SIT allergen composition change in number of allergens mixed by age.


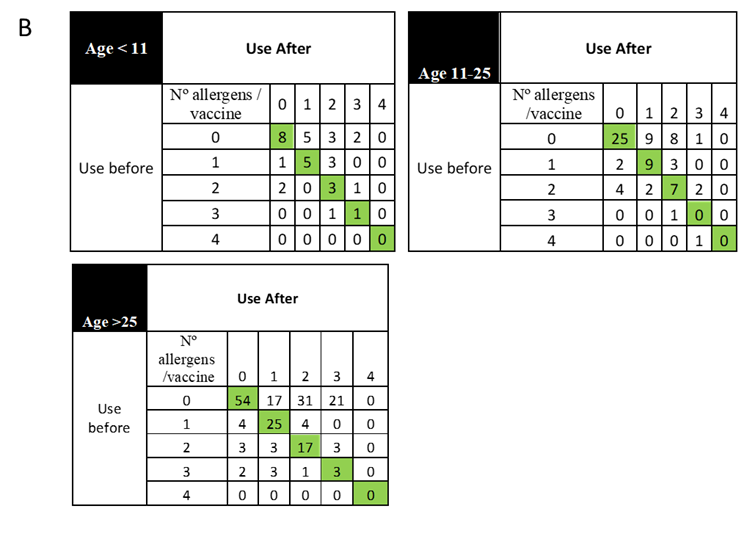


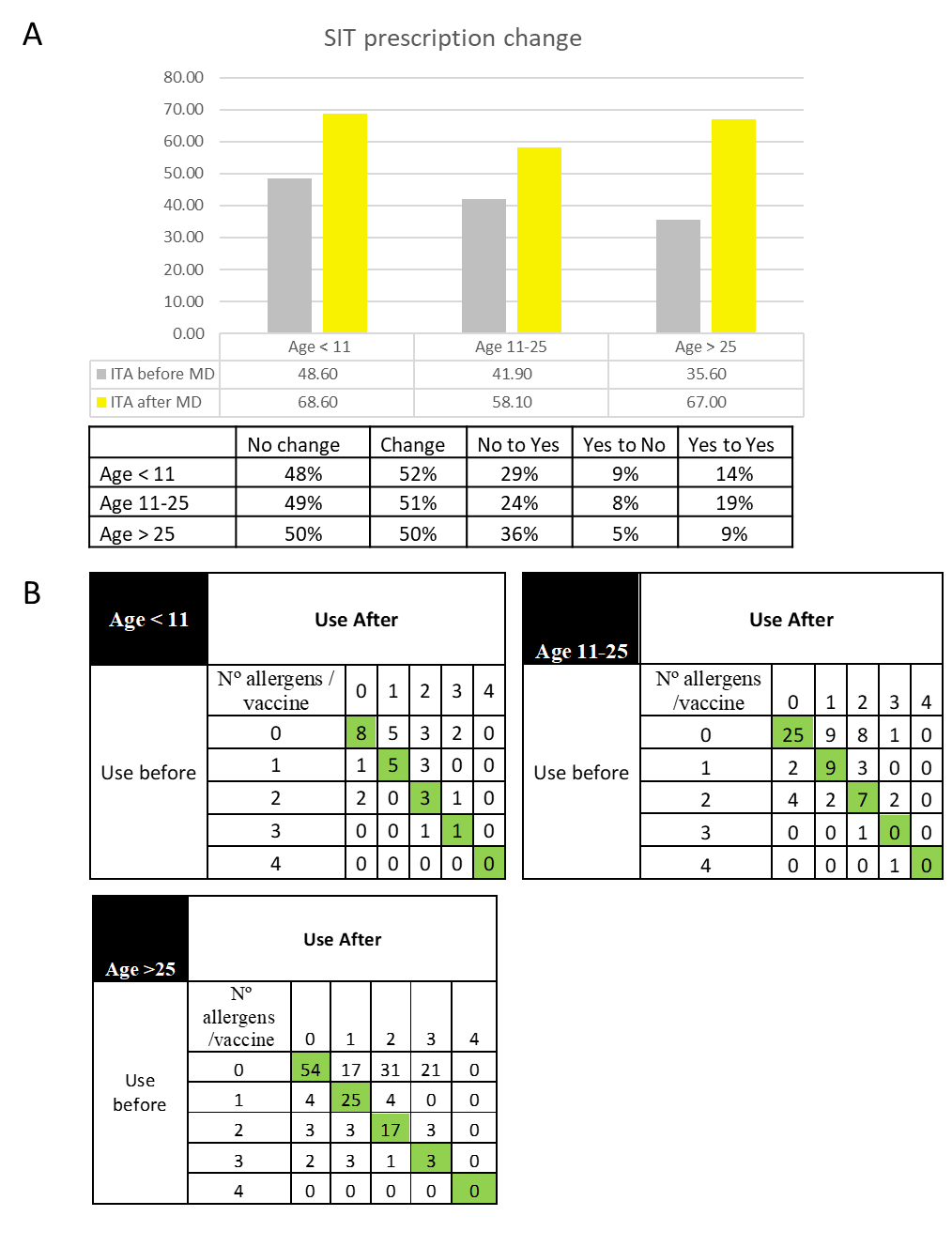


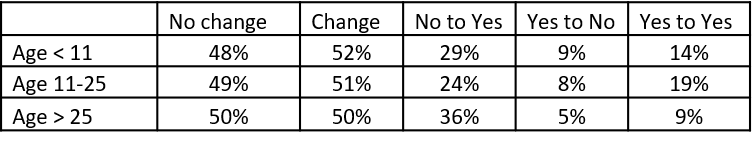


A

B

Supplementary table 4. Study of the correlation between asthma/rhinoconjunctivitis severity and the degree of sensitization to molecular components.

| **Disease** | **Pollen** | **Disease severity** | **N** | **Mean** | **Sd** | **IC95-Low** | **IC95-Up** | **Mín.** | **Median** | **Max.** | **IQR** | **P value** |
| --- | --- | --- | --- | --- | --- | --- | --- | --- | --- | --- | --- | --- |
| **RC** | **Phl p 1** | Mild | 128 | 7.7 | 25.16 | 3.3 | 12.1 | 0 | 0.08 | 238.91 | 6.74 | 0.265 |
|  |  | Moderate | 148 | 12.1 | 23.93 | 8.21 | 15.98 | 0 | 0.81 | 145.83 | 11.62 |  |
|  | **Cup a 1** | Mild | 128 | 6.93 | 17.1 | 3.94 | 9.92 | 0 | 0.3 | 107.21 | 5.8 | 0.282 |
|  |  | Moderate | 148 | 9.79 | 18.89 | 6.72 | 12.86 | 0 | 2.31 | 118.47 | 10.08 |  |
|  | **Ole e 1** | Mild | 128 | 7.94 | 26.87 | 3.24 | 12.63 | 0 | 0.61 | 226.16 | 3.4 | 0.053 |
|  |  | Moderate | 148 | 17.81 | 39.87 | 11.34 | 24.29 | 0 | 1.48 | 287.29 | 13.3 |  |
|  | **Pla a 1** | Mild | 128 | 3.1 | 10.96 | 1.18 | 5.02 | 0 | 0 | 99.01 | 0.55 | 0.789 |
|  |  | Moderate | 148 | 4 | 11.49 | 2.13 | 5.87 | 0 | 0 | 70.1 | 0 |  |
|  | **Art v 1** | Mild | 128 | 0.17 | 0.69 | 0.05 | 0.29 | 0 | 0 | 5.81 | 0 | 0.068 |
|  |  | Moderate | 148 | 0.67 | 2.4 | 0.28 | 1.06 | 0 | 0 | 16 | 0 |  |
|  | **Par j 2** | Mild | 128 | 4.05 | 10.27 | 2.25 | 5.84 | 0 | 0 | 72.25 | 2.87 | 0.053 |
|  |  | Moderate | 148 | 10.08 | 27.01 | 5.69 | 14.47 | 0 | 0 | 160.36 | 5.74 |  |
|  | **Sal k 1** | Mild | 128 | 0.77 | 3.97 | 0.08 | 1.47 | 0 | 0 | 28.68 | 0 | 0.032 |
|  |  | Moderate | 148 | 2.93 | 8.94 | 1.48 | 4.38 | 0 | 0 | 57.64 | 0 |  |
|  | **Bet v 1** | Mild | 128 | 0.17 | 0.92 | 0.01 | 0.33 | 0 | 0 | 8.6 | 0 | 0.188 |
|  |  | Moderate | 148 | 0.31 | 1.71 | 0.03 | 0.58 | 0 | 0 | 11.59 | 0 |  |
|  | **Phl p 1** | Mild | 99 | 7 | 16.51 | 3.7 | 10.29 | 0 | 0.21 | 126.27 | 6.29 | 0.138 |
| **Asthma** |  | Moderate | 26 | 12.78 | 21.31 | 4.18 | 21.39 | 0 | 3.49 | 73.94 | 12.41 |  |
|  | **Cup a 1** | Mild | 99 | 8.47 | 16.49 | 5.18 | 11.75 | 0 | 1.59 | 87.25 | 8.51 | 0.583 |
|  |  | Moderate | 26 | 6.55 | 12.87 | 1.35 | 11.74 | 0 | 1.15 | 61.66 | 8.13 |  |
|  | **Ole e 1** | Mild | 99 | 13.98 | 30.84 | 7.83 | 20.13 | 0 | 1.42 | 173.21 | 10.47 | 0.352 |
|  |  | Moderate | 26 | 8.18 | 13.38 | 2.77 | 13.58 | 0 | 1.93 | 50.16 | 7.98 |  |
|  | **Pla a 1** | Mild | 99 | 4 | 11.34 | 1.74 | 6.26 | 0 | 0 | 61.1 | 0.53 | 0.163 |
|  |  | Moderate | 26 | 0.84 | 2.86 | -0.32 | 1.99 | 0 | 0 | 13.21 | 0 |  |
|  | **Art v 1** | Mild | 99 | 0.3 | 1.65 | -0.03 | 0.63 | 0 | 0 | 16 | 0 | 0.157 |
|  |  | Moderate | 26 | 0.9 | 2.72 | -0.2 | 2 | 0 | 0 | 11.7 | 0 |  |
|  | **Par j 2** | Mild | 99 | 5.09 | 12.69 | 2.55 | 7.62 | 0 | 0 | 87.94 | 4.29 | 0.537 |
|  |  | Moderate | 26 | 7.16 | 22.56 | -1.95 | 16.27 | 0 | 0 | 107.34 | 0 |  |
|  | **Sal k 1** | Mild | 99 | 2.11 | 8.12 | 0.49 | 3.73 | 0 | 0 | 57.64 | 0 | 0.682 |
|  |  | Moderate | 26 | 1.42 | 5.02 | -0.61 | 3.45 | 0 | 0 | 19.22 | 0 |  |
|  | **Bet v 1** | Mild | 99 | 0.36 | 1.8 | 0 | 0.72 | 0 | 0 | 12.67 | 0 | 0.865 |
|  |  | Moderate | 26 | 0.3 | 1.4 | -0.27 | 0.86 | 0 | 0 | 7.13 | 0 |  |

Supplementary table 5. Immunotherapy specific extracts change per patient.

| Immunotherapy Composition | |
| --- | --- |
| Before MD | After MD |
| 11 | 10,11 |
| 11 | 11 |
| 0 | 0 |
| 0 | 0 |
| 8,9,10 | 8,9,10 |
| 8,10,11 | 10,11 |
| 10,11 | 10,11 |
| 0 | 10,11 |
| 0 | 1,10 |
| 0 | 18 |
| 11 | 0 |
| 0 | 1 |
| 0 | 10 |
| 18 | 18 |
| 8,11 | 8,10,11 |
| 18 | 18 |
| 1,18 | 0 |
| 0 | 8,9,10 |
| 22 | 22 |
| 0 | 0 |
| 0 | 0 |
| 0 | 9,10,11 |
| 0 | 22 |
| 0 | 0 |
| 0 | 0 |
| 0 | 0 |
| 1,2,8,11 | 8,9,10 |
| 11 | 11,22 |
| 0 | 0 |
| 1,18 | 1,18 |
| 0 | 0 |
| 9,10 | 1,9 |
| 0 | 0 |
| 1,10 | 0 |
| 0 | 22 |
| 0 | 0 |
| 0 | 10,11 |
| 0 | 9 |
| 18 | 18 |
| 0 | 0 |
| 18 | 18 |
| 0 | 0 |
| 8,11 | 10,11 |
| 0 | 0 |
| 10,11 | 0 |
| 0 | 0 |
| 0 | 1,9 |
| 10 | 9,10 |
| 1,24 | 1,24 |
| 18 | 18 |
| 0 | 9 |
| 10,11 | 9,10,11 |
| 11,18 | 11,18 |
| 0 | 11 |
| 1,11 | 0 |
| 0 | 1,18 |
| 1,11 | 10,11 |
| 10 | 18 |
| 0 | 0 |
| 0 | 18 |
| 8 | 8 |
| 1,10 | 1,3 |
| 1,8,9 | 1,9 |
| 0 | 0 |
| 0 | 10,11 |
| 8 | 0 |
| 1 | 1 |
| 0 | 0 |
| 0 | 0 |
| 1,8 | 1 |
| 10,11 | 10 |
| 10 | 10 |
| 2,8 | 0 |
| 0 | 0 |
| 0 | 0 |
| 0 | 0 |
| 0 | 0 |
| 0 | 0 |
| 18 | 18 |
| 0 | 9 |
| 0 | 8,9 |
| 0 | 8,10,11 |
| 0 | 1,9 |
| 0 | 0 |
| 8,10 | 8,9,10 |
| 0 | 1,10 |
| 0 | 0 |
| 10 | 10 |
| 0 | 6 |
| 8,9,10 | 10 |
| 0 | 10,11 |
| 0 | 0 |
| 0 | 0 |
| 0 | 18 |
| 0 | 9,11 |
| 0 | 0 |
| 0 | 0 |
| 0 | 0 |
| 0 | 1 |
| 0 | 0 |
| 0 | 1 |
| 0 | 0 |
| 0 | 0 |
| 8 | 0 |
| 0 | 0 |
| 0 | 5,6,11 |
| 0 | 1,10 |
| 18 | 18 |
| 0 | 0 |
| 0 | 9,10,11 |
| 8,10,11 | 0 |
| 0 | 1,9,11 |
| 1 | 1 |
| 8 | 11 |
| 1,11 | 1,11 |
| 0 | 8,9,11 |
| 1,10,11 | 11 |
| 0 | 3,11 |
| 1,9,11 | 1,9,11 |
| 0 | 18 |
| 0 | 10,11 |
| 0 | 10,11 |
| 10,11 | 10,11 |
| 0 | 10,11 |
| 0 | 10,11 |
| 10,11 | 0 |
| 1,10 | 1,10 |
| 0 | 9,11 |
| 0 | 9,10,11 |
| 0 | 9,10,11 |
| 0 | 9,11 |
| 0 | 1,8,9 |
| 0 | 11 |
| 9,10 | 9,10 |
| 0 | 10 |
| 0 | 1,2 |
| 0 | 11 |
| 0 | 0 |
| 0 | 1,9,10 |
| 0 | 10,11 |
| 1 | 1,8 |
| 1 | 1 |
| 1,11 | 1,11 |
| 1,10 | 1,10 |
| 1,2,11 | 1,11 |
| 1,11 | 1,9,11 |
| 1,11 | 1,9,11 |
| 0 | 0 |
| 8,11 | 11 |
| 0 | 0 |
| 8 | 0 |
| 0 | 1,9,10 |
| 10 | 10 |
| 0 | 3,9 |
| 0 | 0 |
| 8 | 8,9 |
| 8,11 | 8,11 |
| 0 | 0 |
| 8,9,10 | 8,9,10 |
| 0 | 0 |
| 0 | 0 |
| 11 | 11 |
| 8 | 8 |
| 1 | 1 |
| 1 | 1 |
| 0 | 0 |
| 1,11 | 1,8,11 |
| 10,11 | 11 |
| 0 | 0 |
| 10 | 10 |
| 0 | 0 |
| 0 | 0 |
| 0 | 1 |
| 0 | 9,10 |
| 2,8 | 0 |
| 1,9 | 1,9 |
| 0 | 1,10,11 |
| 0 | 0 |
| 10,11 | 0 |
| 0 | 0 |
| 0 | 1,11 |
| 0 | 1,11 |
| 0 | 8,9 |
| 0 | 0 |
| 1,10 | 1,10 |
| 0 | 1 |
| 0 | 10,11 |
| 0 | 1,9 |
| 0 | 0 |
| 8,11 | 8,11 |
| 10 | 10 |
| 0 | 0 |
| 9,11 | 9 |
| 8 | 9 |
| 0 | 8,11 |
| 0 | 9,10,11 |
| 0 | 9,11 |
| 0 | 1,11 |
| 0 | 11 |
| 0 | 10 |
| 1 | 1 |
| 10,11 | 10,11 |
| 9 | 9,10 |
| 0 | 0 |
| 0 | 0 |
| 0 | 0 |
| 0 | 10,11 |
| 0 | 0 |
| 0 | 1,9 |
| 0 | 0 |
| 0 | 1,9,11 |
| 0 | 0 |
| 0 | 1,8,10 |
| 9 | 9 |
| 0 | 1,8,9 |
| 0 | 1,9,11 |
| 0 | 0 |
| 1,10 | 1,10 |
| 0 | 1,9,10 |
| 0 | 1,5 |
| 0 | 0 |
| 0 | 1,8 |
| 1 | 1 |
| 8,9,10 | 9 |
| 0 | 0 |
| 0 | 8,10,11 |
| 0 | 8,9,10 |
| 8 | 8 |
| 0 | 0 |
| 0 | 1 |
| 8 | 0 |
| 9,10 | 9,10 |
| 0 | 1,2,9 |
| 0 | 10 |
| 0 | 1,8 |
| 1 | 1 |
| 0 | 0 |
| 0 | 9,11 |
| 0 | 0 |
| 0 | 0 |
| 0 | 2,10, 6 |
| 0 | 0 |
| 8 | 8,11 |
| 1,8,10 | 1,8,10 |

Legend:

| Code | Name |
| --- | --- |
| 1 | *Parietaria judaica* |
| 2 | *Artemisia vulgaris* |
| 3 | *Salsola kali* |
| 4 | *Chenopodium album* |
| 5 | *Plantago lanceolata* |
| 6 | *Betula verrucosa* |
| 7 | *Corylus avellana* |
| 8 | *Platanus acerifolia* |
| 9 | *Cupressus arizonica* |
| 10 | *Olea europea* |
| 11 | Grass |
| 18 | HDM |
| 22 | *Alternaria alternate* |
| 24 | Horse |
